# The Uptake and Release of Amino Acids by *Staphylococcus aureus* at Mid-Exponential and Stationary Phases and Their Corresponding Responses to Changes in Temperature, pH and Osmolality

**DOI:** 10.3389/fmicb.2019.03059

**Published:** 2020-01-23

**Authors:** Mousa M. Alreshidi, R. Hugh Dunstan, Margaret M. Macdonald, Johan Gottfries, Tim K. Roberts

**Affiliations:** ^1^Department of Biology, College of Sciences, University of Ha’il, Ha’il, Saudi Arabia; ^2^Metabolic Research Group, Faculty of Science, School of Environmental and Life Sciences, University Drive, Callaghan, NSW, Australia; ^3^Department of Chemistry, University of Gothenburg, Gothenburg, Sweden

**Keywords:** amino acid uptake, *S. aureus*, valine, stress response and adaptation, phenotypic shift

## Abstract

*Staphylococcus aureus* is an important pathogen that is associated with nosocomial infections, as well as food poisoning. This bacterium is resistant to antimicrobial agents and can survive in a wide range of environmental conditions. The aim of this study was to measure the uptake and release of amino acids by *S. aureus* at mid-exponential and stationary phases of growth following exposure to a combination of conditions including variations in temperature, pH and NaCl. Bacterial cells were grown up to mid-exponential and stationary phases in tryptic soy broth (TSB), where the supernatants were collected for analyses of amino acids to determine the uptake and release characteristics. The uptake/release of amino acids was estimated by subtracting the initial levels of the free amino acids in the media from those measured at mid-exponential and stationary phases of growth. When cells were grown at ideal conditions, the analyses revealed that significant uptake of amino acids had occurred by stationary phase compared with the mid-exponential phase. A substantial release of valine and tyrosine into the external media was observed by cells at stationary phase. At both phases, the uptake and release patterns were significantly different between cells grown under ideal control conditions, when compared with those grown under various combinations of sub-optimal environmental conditions. The analyses of the supernatants harvested from controls and treatment groups at exponential phase indicated that the total uptake of amino acids was reduced approximately five times by cells grown with addition of 2.5% NaCl or with pH6 at 35°C, and 2-fold by cells grown at pH8 at 35°C. However, the final quantities of amino acids taken up by cells grown to stationary phase did not significantly alter between control and treated samples. Valine was found to be the most abundant amino acid that was significantly released into the media at stationary phase by both control and treated samples. It was evident that diverse environmental conditions resulted in differential patterns of amino acid uptake and release during adaptation to designated conditions.

## Introduction

*Staphylococcus aureus* is an important pathogen capable of causing a wide range of infections from mild skin and food-borne intoxications to life-threatening sepsis and endocarditis (Josse et al., 2017). Staphylococci have been shown to alter their cytoplasmic composition in response to alterations in the environment such as variation in temperatures, pH and osmolality (Alreshidi et al., 2013; Onyango and Alreshidi, 2018). One of the key areas of interest in food processing industries is the study of bacterial adaptation in harsh environmental conditions, as the understanding of bacterial adaptation and survival mechanisms would pave ways to reduce food-borne illnesses (Zeaki et al., 2014). Survival of bacteria relies on their ability to induce the optimum metabolic homeostasis required for growth in the altered environments (Wolf et al., 2008; Solis et al., 2014; Alreshidi et al., 2015; Onyango and Alreshidi, 2018). Adjustments to environmental influences can lead to significant changes in morphological structure, including cell/colony sizes, and induce the production of biofilms (Zhu et al., 2007; Onyango et al., 2012; Onyango et al., 2013; Crompton et al., 2014).

Small colony variants (SCVs) can be induced as a response to altered environmental conditions including variations in pH, osmotic pressure, temperatures and exposures to antimicrobial agents, etc. (von Eiff, 2008; Onyango et al., 2012; Onyango et al., 2013; Proctor et al., 2014). The occurrence of SCVs have been observed in medical samples, animals (Tkadlec et al., 2015) and even food (Li et al., 2016). SCVs are a slow-growing sub-population of bacteria that are characterized with decreased metabolic activity and increased resistance to antimicrobial agents (von Eiff et al., 2000). SCVs of *S. aureus* were found to exhibit enhanced biofilm formation in comparison to their corresponding wild-type strains (Singh et al., 2010). The exposure of *S. aureus* to variations in NaCl concentrations led to increased production of biofilm, with concomitant increase in the expression of genes associated with biofilm such as the *icaA* gene. Thus, the use of NaCl in processed and canned foods may increase biofilm production of *S. aureus* (Lee et al., 2014). Further, biofilm formation can occur in response to other non-ideal growth conditions such as exposure to alkaline environments (Jones et al., 2015; Jaishankar and Srivastava, 2017). Both SCV phenotypes and biofilm greatly contribute to the profound resistance and stable adhesion, which eventually assist the bacteria to survive under changed environmental conditions (Yao et al., 2005; Li et al., 2016).

A prior investigation has shown that selective amino acids were taken up by the biofilm cultures of *S. aureus* when compared with their corresponding planktonic counterparts (Ammons et al., 2014). Hence, it was hypothesized that amino acid uptake is essential for staphylococcal biofilm development and adjustment to changes in pH (Beenken et al., 2004; Zhu et al., 2007). Another study showed that limited proline in the media impaired staphylococcal growth and survival (Schwan et al., 2004). likewise, combinations of subtle environmental parameters led to substantial modifications in amino acid uptake profiles associated with significant patterns of cytoplasmic amino acids (Murphy et al., 2018). Similarly, significant changes in cytoplasmic amino acid compositions of *S. aureus* in response to variation in pH, temperature and osmotic pressure were observed (Alreshidi et al., 2016). Exometabolome analysis of different strains of *S. aureus* exhibited substantial differences in the uptake of extracellular metabolites (Dorries and Lalk, 2013) and cells grown in culture with limited supplies of glucose also altered their uptake of metabolites (Liebeke et al., 2011). This was proposed as an adaptive mechanism by the bacteria to utilize amino acids for energy and alternative resources to support growth based on nutrient availabilities from the surrounding environment (Zhu et al., 2007; Stipetic et al., 2016). To date, the studies have demonstrated that the exposure of *S. aureus* to subtle variations in temperature, pH and NaCl resulted in significant alterations in cytoplasmic amino acids, proteins and external morphological structures compared with bacterial cells grown under normal conditions (Crompton et al., 2014; Wehrli et al., 2014; Alreshidi et al., 2015; Alreshidi et al., 2016; Alreshidi et al., 2019). It has also been shown that *S. aureus* consumed a significant amount of glycine when incubated in minimal media supplemented with 5% NaCl compared to the cells grown in normal conditions (Alreshidi et al., 2019). The present study investigated whether *S. aureus* could adjust the amino acid uptake profiles from growth media in response to varied environmental parameters (pH, temperature and NaCl) when grown to mid-exponential and stationary phases. It was hypothesized that *S. aureus* would require a rapid adaptation to changes in the temperature, pH and osmolality that require simultaneous rapid alterations in the demand of biosynthesis of metabolites and proteins essential for bacterial survival and growth. Therefore, to do so, the bacterium would alter its amino acid uptake profiles to obtain the optimum metabolism required for adaptation and survival following exposure to changes in the environmental conditions.

## Materials and Methods

### Bacterial Growth Conditions and Experimental Design

The strain of *S. aureus* used in current study was isolated from a patient who hospitalized due to prolonged muscle pain (Butt et al., 1998). This bacterial strain used in the following studies to investigate metabolic and proteomic changes to environmental stresses (Alreshidi et al., 2015, 2016, 2019). The bacterium has been maintained as culture stock on horse blood agar (HBA) and preserved appropriately on sterile glass beads at −80°C with a consistent sub-culturing to preserve cells viability. The identity of the bacteria strain was confirmed regularly using API^TM^ Staph biochemistry and through PCR (Brown et al., 2001).

*Staphylococcus aureus* was cultured in a sub-optimal of environmental conditions for analyses of amino acid uptake by *S. aureus* following growth in tryptic soy broth media (TSB) (Oxoid Ltd-Australia). The reference control included cells grown under ideal conditions of pH7 at 37°C with no added NaCl in tryptic soy broth medium (TSB) and three sets of experimental conditions were applied with (1) pH7 at 37°C with NaCl added (2.5%) in TSB; (2) 35°C and pH6 with no added NaCl in TSB; and (3) 35°C and pH8 with no added NaCl in TSB.

An overnight starter culture (50 ml) of *S. aureus* was grown for 16 h in Tryptic Soy Broth (TSB) at 37°C with constant agitation (120 rpm) to be used as an inoculum for the growth experiments. Replicates of each condition containing 95 ml TSB culture media were inoculated with 5 ml of overnight culture (OD_600_ = 0.1 ≈ 10^8^ CFU/ml)in 500 ml conical flasks and incubated at 37°C with constant agitation (120 rpm). Growth was monitored by aseptically monitoring the absorbance at 600 nm of the cultures to estimate cell numbers, so that the cultures could be harvested at either mid-exponential or stationary phases. Culture supernatants were generated by centrifugation at 6,000 × *g* for 25 min and the supernatants removed for subsequent analyses.

### Amino Acid Uptake Assay

Preliminary analyses of the tryptic soy broth media (TSB) were performed to determine that it contained 23 amino acids (listed in Table 1) and their derivatives, which were available for direct uptake by the bacterial cells. Utilization of these amino acids by the bacteria for growth was assessed by collecting five ml of *S. aureus* cultures grown in TSB media at both mid-exponential and stationary phases. The collected supernatants were filtered through a Millex^®^ membrane filter (0.22 mμ) and stored at −20°C for further analysis. 100 μl of sterile TSB media and the filtered supernatants harvested at mid-exponential phase were diluted with 300 μl of sterile Milli-Q water, and the 100 μl harvested at stationary phase was diluted with 100 μl with Milli-Q water. The dilution was done to avoid overwhelming the analytical capacity of the gas chromatography column. An aliquot of 100 μl of diluted supernatants was then processed and analyzed using a commercial analytical kit (Phenomenex^®^ EZ: faast^TM^). The technique was processed according to the manufacturer’s instructions. The derivatized amino acids were then analyzed using an Agilent gas chromatograph (Hewlett Packard HP 6890 series) coupled with flame ionization detector (GC-FID) which was calibrated to measure more than 40 amino acid metabolites as previously described (Onyango et al., 2012). The injection volume was 2 μl with splitless mode and flow rate of the carrier gas (Helium) was 0.5 ml/min. Nor-valine was used as an internal standard to calculate the concentrations of amino acids present in the sample as nmol/100 μl. The results were normalized by multiplied by dilution factors.

**TABLE 1:** Comparisons of the quantities of amino acids taken up or released by *S. aureus* at mid-exponential and stationary phases, during growth under optimal conditions (mean ± SD, *P* < 0.05).

	**Amino acids**	**Mid-exponential phase (nmole/100 μL) *n* = 4 biological replicates**		**Stationary phase (nmole/100 μL) *n* = 3 biological replicates**	
			
		**Taken up**	***Released***	**Taken up**	***Released***
Amino acids with significantly higher levels of	LYS	74.2 ± 14.9		154 ± 4.6	
uptake measured at the stationary phase compared	LEU	70.9 ± 28.8		292 ± 1.2	
with mid-exponential phase	SER	56.3 ± 5.8		135 ± 0.0	
	PHE	41.9 ± 11.6		63.8 ± 2.4	
	GLU	34.2 ± 3.8		98.8 ± 1.5	
	THR	29.6 ± 3.4		86.9 ± 0.1	
	ALA	28.5 ± 4.5		108 ± 0.2	
	ASN	27.3 ± 2.0		52.0 ± 0.3	
	GLY	25.9 ± 1.9		50.4 ± 0.0	
	HIS	5.2 ± 1.5		18.3 ± 0.1	
	PRO	3.2 ± 0.9		14.6 ± 0.0	
	AAA		1.5 ± 2.5	6.3 ± 0.0	
	GLN		5.0 ± 6.7	12.7 ± 7.2	
	ORN		0.45 ± 0.4	1.3 ± 0.5	
Amino acids with significantly lower levels of uptake	ILE	23.0 ± 5.5		11.8 ± 4.6	
measured at the stationary phase compared with	TRP	10.7 ± 2.9		5.5 ± 2.0	
mid-exponential phase					
Amino acids which did not show any further	MET	18.8 ± 3.5		23.8 ± 1.4	
significant up-take after mid-exponential phase	ASP	10.7 ± 1.8		8.8 ± 1.2	
	TPR	4.6 ± 2.1		6.6 ± 0.0	
	HYP	4.1 ± 0.0		2.8 ± 0.9	
	ABA	2.4 ± 1.9		2.4 ± 0.3	
Amino acids that were taken up by mid-exponential	VAL	30.0 ± 8.4			81.8 ± 6.5
phase and then released by stationary phase	TYR	19.1 ± 4.3			17.0 ± 3.7

*The amino acids have been ranked in the order of their magnitudes of uptake measured at the mid-exponential phase of growth. Amino acid uptake/release was calculated by subtracting the initial concentrations of amino acids in TSB from those evaluated at mid-exponential and stationary phase.*

### Statistical Analyses

The data generated from gas chromatography and flame ionization (GC−FID) were evaluated via ANOVA to detect the uptake of amino acids that were significantly altered following growth in variations in temperature, pH and NaCl (Statistica, TIBCO Software Inc. [2017], data analysis software system, version 13)^1^. Principal component analyses (PCA) were also performed. The data were subjected to mean centering and unit variance scaling before PCA calculations. The model complexity and validity were assessed by cross validation as applied in the software.

## Results

### Amino Acid Uptake/Release at Mid-Exponential and Stationary Phases by Cells Grown Under Ideal Conditions

Cultures of *S. aureus* were grown in TSB to mid-exponential and stationary phases of growth under optimal conditions (pH7, 37°C and no added NaCl). The analyses of the amino acid compositions in cultures supernatants revealed the presence of 23 amino acids and amino acid derivatives (Table 1). The analysis indicated that lysine, leucine and serine were taken up in the greatest quantities at both phases, and it was noted that the uptake of leucine was 4 times higher at stationary phase compared with the exponential phase, whereas serine and lysine were 2.4 and 2-times higher respectively. Valine and tyrosine were taken up during growth to mid-exponential phase of growth, but it was evident that by stationary phase, these amino acids had been released back into the external medium to yield a final concentration higher than present in the original TSB medium. The uptake of five amino acids did not significantly change between phases, including aspartic acid, tryptophan and methionine, despite their high quantity in the initial growth media. Small quantities of glutamine were released into the medium by mid-exponential phase, but some uptake was evident by stationary phase. Isoleucine and tryptophan were taken up by mid exponential phase, but then appeared to be released back into the medium by the stationary phase.

**FIGURE 1:**
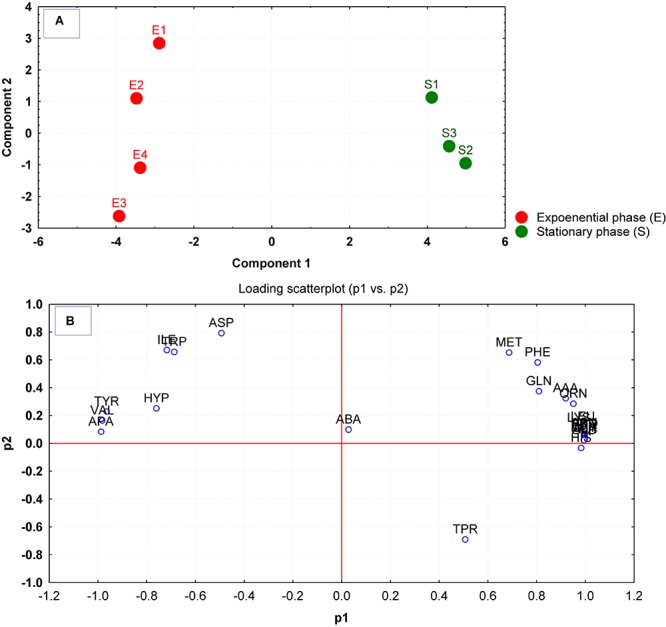
**(A)** Principal component analysis (PCA) scores generated from the amino acid compositions of the culture supernatants after growth were plotted for each replicate measured at the mid-exponential (E1–E4) and stationary phase (S1–S4). **(B)** The corresponding loading scatter plot of the amino acids (abbreviations from Table 1) indicates the influence of amino acid uptake/release on the clustering and separation on the PCA plot.

The differences in the uptake of amino acids from the culture supernatants were additionally investigated through principal component analysis (PCA). As per the PCA plot, the biological replicates for each phase were tightly grouped, but well resolved by the uptake/release of amino acids (Figure 1A). The samples of amino acid uptake/release estimated at the mid-exponential phase were spread along with the negative component 1 while the replicates of amino acid uptake from culture supernatants measured at the stationary phase of growth were distributed at the positive side of component 1 (Figure 1A). The PCA loading scatterplot analysis indicated that distinct amino acid uptake/release contributed substantially to the discrimination of growth phases (Figure 1B).

### Amino Acids Uptake and Release Analysis at Mid-Exponential Phase

Cultures of *S. aureus* (4 replicates each) were grown to mid-exponential phase of growth under various combinations of sub-optimal environmental conditions with temperature ranging from 35 to 37°C, pH 6–8 and higher NaCl (0–2.5%). The supernatants were collected at mid-exponential phase of growth to analyze the uptake/release of amino acids. The amino acid uptake and release determined in each of the replicates were reproducible within each treatment group with specific changes in the uptake/release of amino acids (Table 2). On analyzing the uptake of the amino acids in controls, lysine, leucine, and serine were found to be the major amino acids extracted from the culture media, accounting for 14.5, 13.9, and 11% of total amino acids consumed, respectively (Table 2). However, when bacterial cells were grown under the alternative environmental conditions designated B–D in Table 2, the uptake of lysine was greatly diminished and leucine was actually released into the external medium in treatments B and C. Serine and glycine became the major amino acids taken up from the media in treatments B–D. Various specific alterations in amino acid uptake and release associated with the different environmental conditions were evident in Table 2. At pH8 and 35°C with no added NaCl, glutamic acid was the second most consumed amino acid after serine. The total quantities of amino acids taken up was 3.5 times lower when bacterial cells were grown in the presence of 2.5% NaCl in treatment B. Reduction of temperature to 35°C under more acidic (C) or alkali (D) conditions resulted in a 3.3 and 1.8-fold decline in the total uptake of amino acids, respectively.

**TABLE 2:** Comparisons of the quantities of amino acids taken up or released by *S. aureus* at the mid-exponential phase of growth under optimal conditions **(A)** compared with growth under sub-optimal environmental conditions **(B–D)**.

**Amino**	**(A) Control pH7 at 37°C with no**		**(B) pH7 at 37°C with 2.5%**		**(C) pH6 and 35°C with no**		**(D) pH8 at 35°C with no**	
**acids**	**added NaCl. nmole/100 μL**		**NaCl added. nmole/100 μL**		**added NaCl nmole/100 μL**		**added NaCl nmole/100 μL**	
				
	**Taken up**	**Released**	**Taken up**	**Released**	**Taken up**	**Released**	**Taken up**	**Released**
LYS	74.2 ± 14.9		17.0 ± 37.5*		2.6 ± 12.2*		15.1 ± 17.3*	
LEU	70.9 ± 28.8			24.0 ± 34.1*	17.1 ± 21.2*			6.4 ± 27.4
SER	56.3 ± 5.8		26.9 ± 5.9*		26.6 ± 4.4*		61.6 ± 4.4	
PHE	41.9 ± 11.6		1.4 ± 16.7*		10.4 ± 5.1*		11.3 ± 11.2*	
GLU	34.2 ± 3.8		9.0 ± 4.00*			0.8 ± 5.6*	34.0 ± 3.6	
VAL	30.0 ± 8.4			7.0 ± 12.4*	1.4 ± 7.9*		3.0 ± 7.2*	
THR	29.6 ± 3.4		15.2 ± 5.4*		18.2 ± 5.0*		29.2 ± 2.8	
ALA	28.5 ± 4.5		6.5 ± 7.8*		6.7 ± 4.6*		15.2 ± 5.2*	
ASN	27.3 ± 2.0		16.2 ± 2.9*		14.2 ± 2.3*		28.9 ± 1.4	
GLY	25.9 ± 1.9		25.9 ± 1.6		25.0 ± 1.7		33.9 ± 1.2*	
ILE	23.0 ± 5.5			*0*.5 ± 5.0*	2.2 ± 5.6*		13.2 ± 4.7*	
TYP	19.1 ± 4.3		2.1 ± 5.8*		1.7 ± 3.9*			0.03 ± 4.9*
MET	18.8 ± 3.5		4.2 ± 5.6*		2.3 ± 6.2*		9.6 ± 3.5*	
ASP	10.7 ± 1.8		7.3 ± 0.9*		9.4 ± 2.3*		11.2 ± 2.0*	
TRP	10.7 ± 2.9							
HIS	5.2 ± 1.5		2.2 ± 1.4*		0.4 ± 1.3*		0.5 ± 1.2*	
TPR	4.6 ± 2.1		1.8 ± 0.4*		1.9 ± 0.67*		0.5 ± 0.4*	
HYP	4.1 ± 0.0		0.7 ± 1.6*		0.2 ± 0.31*		3.7 ± 1.6	
PRO	3.2 ± 0.9		7.1 ± 0.4*		2.1 ± 0.65		3.0 ± 0.7	
ABA	2.4 ± 1.9		3.4 ± 0.2		2.0 ± 0.11		0.9 ± 0.2	
GLN		5.0 ± 6.7		24.8 ± 11.8*		8.2 ± 3.4		14.6 ± 6.0
AAA		1.5 ± 2.5		5.8 ± 3.0		3.6 ± 1.4		6.0 ± 2.2
ORN		0.45 ± 0.4		2.4 ± 0.7		0.5 ± 0.2		0.1 ± 0.5

The amino acids have been ranked in the order of their magnitudes of uptake measured at ideal growth conditions (mean ± SD, *P* < 0.05). *Indicates amino acids are significant.

The total quantities of amino acids released was almost six times higher when bacterial cells were exposed to 2.5% NaCl whereas 4.4 and 2.9 times higher when cells were grown at 35°C under more acidic (C) or alkali (D) conditions, respectively.

**FIGURE 2:**
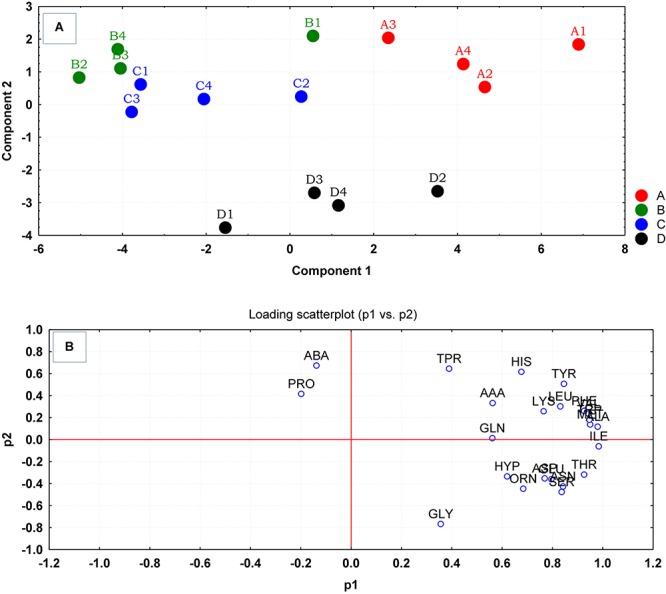
**(A)** Principal component analysis (PCA) scores generated from the amino acid uptake and release data, were plotted for each replicate culture of *S. aureus* grown under ideal conditions (A) and those grown under sub-optimal conditions (B–D) evaluated at the mid-exponential phase of growth. **(B)** The loading scatterplot of the amino acids (abbreviations from Table 1) indicated the influence of variables in PCA plot. The cells were grown under the following conditions: (A1–A4) Control cultures were grown under ideal conditions at pH7 and 37°C with no added NaCl; (B1–B4) Cultures were grown at pH 7 and 37°C with 2.5% NaCl; (C1–C4) Cultures were grown at 35°C and pH6 with no added NaCl; and (D1–D4) Cultures were grown at 35°C and pH8 with no added NaCl.

Multivariate analyses using PCA revealed significant variations between the amino acid compositions of the culture supernatants from the different treatment regimens (Figure 2A). The result of the PCA indicated that the cells grown at pH 8 and 35°C with no added NaCl (D) were the most distinct group from the control and other treatment groups. Groups B and C had more similar but separable amino acid uptake profiles, and groups B–D were very well distinguished from those grown under ideal conditions (A). The loading scatterplot indicates the significance of the variables (amino acids) on PCA plot separation and clustering of the experimental groups (Figure 2B).

### Amino Acids Uptake and Release Analysis at Stationary Phase of Growth

Cultures of *S. aureus* grown under the same environmental conditions A–D, were harvested at the stationary phase of growth to assess the impact on the total uptake and release of amino acids during culture growth. Under ideal growth conditions (A), lysine, leucine, and serine continued to be the most utilized amino acids from the medium (Table 3). This trend was similar for treatments B and D, but treatment C under the alkaline conditions had taken up much lower levels of lysine. The profile of amino acid uptake and release was differential between the treatments with numerous significant differences observed between utilization rates of the amino acids. It was interesting to note that the uptake of alanine and phenylalanine, as the fourth and seventh most utilized amino acids from the medium, was the same across all treatments.

**TABLE 3:** Comparisons of the quantities of amino acids taken up or released by *S. aureus* at the stationary phase of growth under optimal conditions **(A)** compared with growth under sub-optimal environmental conditions **(B–D)**.

**Amino**	**(A) Control pH7 at 37°C with no**		**(B) pH7 at 37°C with 2.5%**		**(C) pH6 and 35°C with no**		**(D) pH8 at 35°C with no**	
**acids**	**added NaCl. nmole/100 μL**		**NaCl added. nmole/100 μL**		**added NaCl nmole/100 μL**		**added NaCl nmole/100 μL**	
				
	**Taken up**	**Released**	**Taken up**	**Released**	**Taken up**	**Released**	**Taken up**	**Released**
LEU	292 ± 1.2		272.9 ± 10.1*		273.9 ± 5.1*		338.4 ± 3.2*	
LYS	154 ± 4.6		132.4 ± 17.9		48.5 ± 14.1*		112.6 ± 0.9*	
SER	135 ± 1.8		116.4 ± 4.4*		141.7 ± 3.6*		147.9 ± 1.1*	
ALA	108 ± 0.2		108.2 ± 0.0		109.4 ± 0.3		115.6 ± 0.1	
GLU	98.8 ± 1.5		85.6 ± 0*		62.3 ± 1.4*		89.4 ± 0.7*	
THR	86.9 ± 0.1		85.5 ± 0.7		108.8 ± 0.9*		100.7 ± 1.0*	
PHE	63.8 ± 2.4		67.7 ± 9.2		71.5 ± 1.8		66.7 ± 0.5	
ASN	52.0 ± 0.3		45.03 ± 0.2*		55.1 ± 0.7*		50.3 ± 0.5*	
GLY	50.4 ± 0.0		51.5 ± 0.0*		55.5 ± 0.3*		53.2 ± 0.17*	
MET	23.8 ± 1.4		24.4 ± 2.13		32.4 ± 0.7*		32.01 ± 0.9*	
HIS	18.3 ± 0.9		29.0 ± 0.1*		26.01 ± 0.4*		22.8 ± 0.17*	
PRO	14.6 ± 1.8		14.2 ± 0.8		15.8 ± 0.7*		12.8 ± 0.9*	
GLN	12.7 ± 7.2		6.5 ± 10.5		19.01 ± 27.2		5.67 ± 22.8	
ILE	11.8 ± 4.6		20.9 ± 2.5*		3.8 ± 2.4*		28.5 ± 1.79*	
ASP	8.8 ± 1.2		11.7 ± 0.9*		29.7 ± 0.2*		28.4 ± 0.6*	
TPR	6.6 ± 0.0		7.8 ± 0		9.4 ± 0.0		6.6 ± 0.0	
AAA	6.3 ± 0.0		8.8 ± 0.0		4.3 ± 0.0		6.7 ± 0.0	
TRP	5.5 ± 2.0		14.1 ± 4.2*		19.4 ± 1.2*		18.4 ± 1.6*	
HYP	2.8 ± 0.9		2.3 ± 0.8		2.7 ± 0.6		2.5 ± 0.9	
ABA	2.4 ± 0.3		4.2 ± 0.4*		5.1 ± 0.0*		3.6 ± 0.3*	
ORN	1.3 ± 0.15			21.16 ± 1.5*		11.1 ± 0.9*	1.2 ± 0.1	
VAL		81.8 ± 6.5		46.69 ± 7.2*		91.3 ± 5.6		76.5 ± 1.6
TYR		17.0 ± 3.7		6.04 ± 3.8*		4.8 ± 3.8*		13.7 ± 3.7

The amino acids have been ranked in the order of their magnitudes of uptake measured at optimal conditions (mean ± SD, *P* < 0.05). *Indicates amino acids were significantly uptaken or released.

**FIGURE 3:**
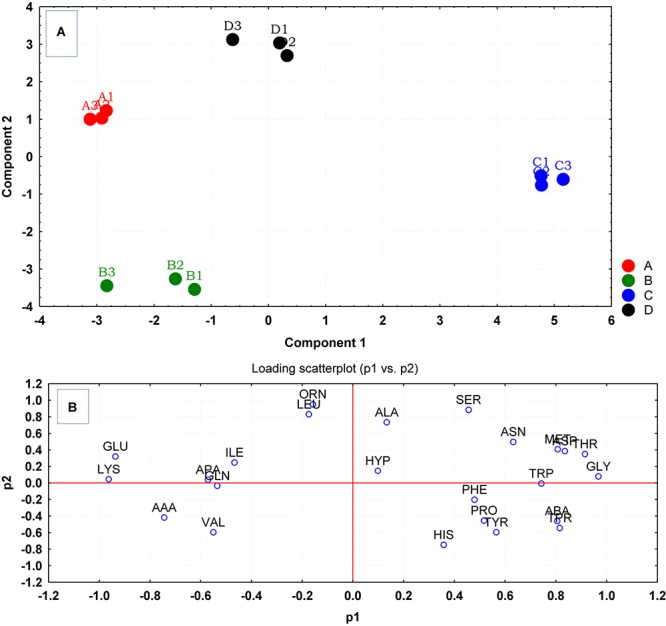
**(A)** Principal component analysis (PCA) scores generated from the amino acid uptake and release data, were plotted each replicate culture of *S. aureus* grown under ideal conditions (A) and those grown under sub-optimal conditions (B–D) evaluated at stationary phase. **(B)** The loading scatterplot of the amino acids (abbreviations from Table 1) indicates the significance of amino acid variables on PCA plot. The cells were grown under the following conditions: (A1–A4) Control cultures were grown under ideal conditions at pH7 and 37°C with no added NaCl; (B1–B4) Cultures were grown at pH 7 and 37°C with 2.5% NaCl; (C1–C4) Cultures were grown at 35°C and pH6 with no added NaCl; and (D1–D4) Cultures were grown at 35°C and pH8 with no added NaCl.

Multivariate analyses using PCA revealed significant variations between the amino acid compositions of the culture supernatants from the different treatment regimens (Figure 3A). Measured amino acids were subjected to multivariate analysis using PCA. The PCA analysis revealed an apparent separation of reference control and treatment samples due to their amino acid uptake and release profiles analyzed at stationary phase of growth. It was also evident from PCA plot that cells gown with lower temperature of 35°C and pH6 (C) had very different amino acid uptake and release profiles compared to equivalent cells grown at pH8 (D). The loading scatterplot corresponding to PCA scores indicates that amino acid uptake/release that contributed to the significance of the clustering/separation observed on the PCA plot, and was unique to the control and treatment regimens (Figure 3B).

### Comparison of Mid-Exponential vs. Stationary Phase Amino Acids Uptake and Release Profiles

**FIGURE 4:**
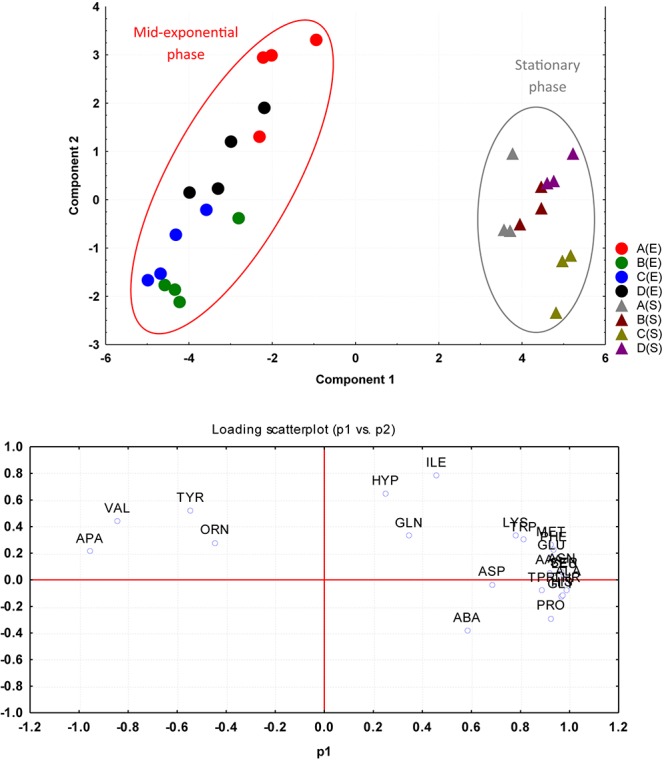
PCA plotted from the four environmental treatment regimens (A–D) were compared between mid-*exponential* (*E series*) and *stationary* (*S series*) phases of growth based on the amino acid uptake and release data. The cells were grown under the following conditions: (A) Control cultures were grown under ideal conditions at pH7 and 37°C with no added NaCl; (B) Cultures were grown at pH 7 and 37°C with 2.5% NaCl; (C) Cultures were grown at 35°C and pH6 with no added NaCl; and (D) Cultures were grown at 35°C and pH8 with no added NaCl.

The amino acid compositions of *S. aureus* culture supernatants from the four environmental treatment regimens (A–D) were compared between the mid-exponential and stationary phases of growth using PCA. The PCA analysis rendered a two-component model as validated by cross-validation (CV). The PCA scores for mid-exponential and stationary phases displayed two clear clusters greatly separated by component 1 scores where the mid-exponential phase had a negative component 1 score and the stationary phase had positive component 1 scores (Figure 4). Explanation of 95% of the data was achieved (i.e., *R*^2^ = 0.70 and *Q*^2^ = 0.64) and the eigenvalue was 16.3 as compared to 2.3 for PC2. The loading scatterplot showed the influence of key variables on the clustering and separation. The stationary phase was characterized via a generally greater level of amino acids taken up from the medium, whilst the mid-exponential phase control cells (A) were characterized by having taken up valine and tyrosine in high quantities before releasing these amino acids back into the medium by the time of assessment at stationary phase.

## Discussion

The outcomes from the current study indicated that the patterns of amino acid uptake and release activities observed in *S. aureus* at the mid-exponential phase of growth were different to those observed at stationary growth phases under optimal growth conditions. It was clear that the demands for amino acids altered throughout the various stages of growth and this varied in relation to variations in the initial environmental conditions involving pH, temperature and osmolality. Amino acids represent an essential resource for sustaining cell integrity and metabolic homeostasis since in addition to their utilization in protein synthesis; they are utilized as precursors for the biosynthesis of primary and secondary metabolites nucleotides, complex lipids and cell wall components. The presence of free amino acids in the complex growth medium facilitates optimal growth capacity under ideal environmental conditions.

The shift from mid-exponential phase to stationary phase is known to involve changes in the environmental conditions such as diminishing levels of nutrients, accumulation of toxic waste products due to metabolism, and changes in pH. Significant uptakes of lysine, leucine and serine at the stationary phase were noted in this study. *S. aureus* possibly consumes these amino acids to balance pH in the cytoplasm that could occur as a result of proton influx (Liebeke et al., 2011). In the stationary phase cultures, the release of large quantities of valine into the external medium was noted. Valine release was observed from *E. coli* biofilm cultures (Valle et al., 2008) and this amino acid was secreted by *S. aureus* COL when it was grown in the eukaryotic cell culture medium (Dorries and Lalk, 2013). Bacteria have been previously reported to produce secondary metabolites and small molecules in the stationary phase of growth (Martin and Liras, 1989; Lopez et al., 2010). These function as signaling molecules to start the process of biofilm induction or inhibiting the growth of other organisms sharing the same environments (Lopez and Kolter, 2010; Santos et al., 2019). At stationary phase, the population density increases significantly, and the production of extracellular molecules that participate in cell-cell communications (quorum sensing) show a high correlation with cell density (Beenken et al., 2004; Resch et al., 2006). Thus the release of some amino acids in the current study could potentially function as a quorum-sense which is involved in the development and maintenance of biofilm. This study showed, as expected, that cells grown to stationary phase progressively consumed additional amounts of amino acids compared after growth to the mid-exponential phase. However, the amino acids were consumed at varying rates, which presumably reflected differential metabolic demands. It is not yet clear why valine and tyrosine would be initially taken up during growth to the mid-exponential phase and then released at high quantities by stationary phase. The levels of both of these amino acids in the external medium at stationary phase indicated that significant quantities of these amino acids were synthesized *de novo*. It has been shown that biofilm cultures consumed and secreted significantly higher amount of metabolites compared to the planktonic cells, suggesting that the increased uptake is a result of high bacterial requirement to synthesize cell wall components and extracellular matrix, which represent a protective mechanism against undesirable conditions present in the stationary phase (Ammons et al., 2014). Lysine uptake increased significantly at the stationary phase, indicating an intermediate proton force in the enhanced uptake of lysine (Liebeke et al., 2011; Stipetic et al., 2016). Interestingly, the uptake of some amino acids did not change significantly between growth phases, possibly pointing out that these amino acids may not be as important as other amino acids, and that these are not part of the adaptive mechanism in the stationary phase.

Exposing *S. aureus* to subtle changes in temperature, pH and osmolality resulted in significant differences in the uptake of amino acids harvested at mid-exponential phase. This indicated that the bacterium had to consume different quantities of amino acids to adapt to changes in the environmental conditions. A previous study exposing the bacterium to the same environmental conditions resulted in different cytoplasmic amino acid profiles (Alreshidi et al., 2016). The reduction in cytoplasmic amino acids is greatly correlated with the reduction of amino acid uptake in this study. This decreased uptake of amino acids is concomitant with reduced amino acids in the cytoplasm, suggesting that the bacterium had undergone a reduced metabolic activity to adapt to the changes in temperature, pH and osmolality (Vandecasteele et al., 2004; Wolf et al., 2008; Tremaroli et al., 2009; Zhu et al., 2009).

It has been shown that the staphylococcal biofilms consumed more arginine and accumulated ornithine in the culture media (Zhu et al., 2007), as was observed for ornithine in the current study. A high production of polysaccharide intercellular adhesin has been associated with reduced tricarboxylic acid activity (Vuong et al., 2005). Hence, this may explain the reduction in amino acid uptake, as it possibly associates with changes in external morphology such as cell size, phenotypic shift and biofilm development (Onyango et al., 2012, 2013). It has been demonstrated that SVCs have a reduced metabolic activity with a decrease of toxins productions. The activity of tricarboxylic acid cycle (TCA) was highly associated with polysaccharide intercellular adhesin (PIA) production in biofilm (Vuong et al., 2005; Sadykov et al., 2008, 2010; Zhu et al., 2009), it showed that active TCA led to repression of PIA production and hence reduced the synthesis of virulence factors (Somerville and Proctor, 2009). Therefore, it has been suggested that the uptake of amino acids would be critical for TCA activation and therefore, pathogenicity. Metabolomic studies have demonstrated that amino acid catabolism is important for the synthesis of intermediates oxaloacetate, oxoglutarate, phosphoenolpyruvate, and pyruvate for TCA and gluconeogenesis (Vuong et al., 2005; Chatterjee et al., 2006; Fleury et al., 2009). On the basis of these results, it has been suggested that the bacterium regularly detecting and adapting to the changes in the external environmental conditions by inducing the best operative and competent phenotypes for survival in stressed environments (de Jonge et al., 1992; Casadevall, 2008; Onyango et al., 2012, 2013).

The assessment of uptake and release patterns of amino acids by *S. aureus* at the stationary phase under various stress-induced conditions revealed remarkable amino acid uptake and release profiles, suggesting that the bacterium alters its amino acid uptake to achieve ideal metabolism related to the external environmental conditions. Surprisingly, the total quantities of amino acids consumed at the stationary phase measurements were very similar under all conditions, yet the individual uptake profiles of amino acids were significantly different. Prior research showed that *Escherichia coli* continued synthesizing proteins at constant rates at stationary phase for several days (Gefen et al., 2014). This may illustrate the higher uptake of amino acids at stationary phase in the present study. The productions of proteins at stationary phase may be linked with the need of the bacterium to combat complex environments present in the stationary phase including altered pH and osmolality. It has been reported that the production of proteins at stationary phase increased by 121% as compared with exponential phase (Ou et al., 2004; Jaishankar and Srivastava, 2017). The release of amino acids in this study may relate to peptidoglycan synthesis and quorum-sensing system (De Kievit et al., 2001; Aliashkevich et al., 2018). Many bacteria release D-amino acids at a stationary stage into the growth medium, which influences the regulation of peptidoglycan components, thickness, and quantity (Aliashkevich et al., 2018). Therefore, the release of these amino acids might be an acclimation mechanism for bacteria to the altered environment (Lam et al., 2009).

PCA analysis of the whole data sets of mid-exponential and stationary phases resulted in two different clusters, one representative of amino acid uptake data analyzed at mid-exponential phase and the second cluster representative of amino acids uptake obtained at stationary phase. This clearly indicated that regardless of alterations in environmental factors applied in this study, the different phases of growth inferred the most influence on uptake characteristics. Stationary phase is considered as a complex environment, and physiologically is comparable to biofilm (Jaishankar and Srivastava, 2017). In stationary phase, extraordinary adjustments take place including morphological, physiological changes and DNA/protein ratio was found to be higher (Nystrom, 2004; Jaishankar and Srivastava, 2017).

## Conclusion

The identification of amino acid uptake profiles that distinguish between control and treatment cultures will have great implications in the translational research for manipulating adaptation and survival of *S. aureus* in response to subtle changes in the environmental conditions. Indeed, the analysis of amino acid uptake/release presented in this study indicated that even within small changes in the environmental conditions, a significant difference in amino acid uptake patterns can be observed. In addition, specific changes in amino acid uptake would provide the basis for adaptation strategies that could lead to evolutionary existence and ideal acclimatization of this bacterium to allow it to capitalize on infective opportunities. It is obvious that certain systematic mechanisms were initiated to survive during the alterations in temperature, pH, and osmolality to acquire optimal metabolism status by consuming specific amino acids. It is thus concluded that these changes in the uptake and release of amino acids are critical for the adaptation processes in response to changes in subtle-environmental conditions.

## Data Availability Statement

The raw data supporting the conclusions of this article will be made available by the authors, without undue reservation, to any qualified researcher.

## Author Contributions

All authors conceived and designed the experiments. MA and RD performed the experiments, analyzed the data, and wrote the manuscript with support from all authors.

## Conflict of Interest

The authors declare that the research was conducted in the absence of any commercial or financial relationships that could be construed as a potential conflict of interest.

## Footnotes

1 http://statistica.io
